# Development of sustainable strontium ferrite graphene nanocomposite for highly effective catalysis and antimicrobial activity

**DOI:** 10.1038/s41598-023-33901-w

**Published:** 2023-04-24

**Authors:** Suranjana V. Mayani, Sandip P. Bhatt, Vishal J. Mayani, Gaurav Sanghvi

**Affiliations:** 1 Department of Chemistry, Marwadi University, Rajkot-Morbi Road, P.O. Gauridad, Rajkot, Gujarat 360003 India; 2 Hansgold ChemDiscovery Center (HCC), Hansgold ChemDiscoveries Pvt. Ltd., Rajkot, Gujarat India; 3 Department of Microbiology, Marwadi University, Rajkot-Morbi Road, P.O. Gauridad, Rajkot, Gujarat 360003 India

**Keywords:** Microbiology, Environmental sciences, Natural hazards, Chemistry, Materials science, Nanoscience and technology

## Abstract

Graphene oxide (GO) has layered structure with carbon atoms that are highly coated with oxygen-containing groups, increasing the interlayer distance while simultaneously making hydrophilic atomic-thick layers. It is exfoliated sheets that only have one or a few layers of carbon atoms. In our work, Strontium Ferrite Graphene Composite (**SF@GOC**) has been synthesized and thoroughly characterized by physico-chemical methods like XRD, FTIR, SEM–EDX, TEM, AFM, TGA and Nitrogen adsorption desorption analysis. A very few catalysts have been manufactured so far that are capable of degrading Eosin-Y and Orange (II) dyes in water by heterogeneous catalytic method. The current study offers an overview of the recyclable nanocomposite **SF@GOC** used in mild reaction conditions to breakdown the hazardous water pollutant dyes Eosin-Y (96.2%) and Orange (II) (98.7%). The leaching experiment has demonstrated that the use of the transition metals strontium and iron have not result in any secondary contamination. Moreover, antibacterial and antifungal assay have been investigated. **SF@GOC** has shown greater activity with bacterial and fungal species while compared with GO. FESEM analysis shows that the bactericidal mechanism for **SF@GOC** is same in both gram-negative bacteria. The difference in the antifungal activity among the candida strains can be correlated with the movement of ions release (slower and faster) of synthesized nanoscrolls in **SF@GOC**. In comparison to previous reports, this new environmentally safe and novel catalyst showed substantial degrading activity. It can also be applied to new multifunctional processes such as in the fields of composite materials, solar energy, heterogeneous catalysis and biomedical applications.

## Introduction

The textile industry uses dyes extensively. The wastewaters from these industries cause significant water pollution. Dyes in wastewater modifies chemically, scavenge dissolved oxygen and terminate aquatic life. Additionally, some dyes and their degradation products may be carcinogenic and/or toxic. So, the wastewaters from the textile industries should be treated before their discharge into environment. The aquatic ecology has been destroyed as a result of the effluent from the dyeing businesses, which contain dangerous and cancer-causing pigments that are harmful to fish, aquatic microbes and animals as well as cause major risks to the ability of photosynthesis for aquatic plants^1^. Treatment of such organic stains in water with small amounts of pollution has long drawn the interest of numerous researchers. The accessible traditional techniques for getting rid of this kind of colour are not suitable for the requirements for current water sources and water quality. Eosin-Y, a red fluorescent dye from the xanthene family, has often been used in a number of applications, including as a biological stain, laser dye, fluorescent study, nanocomposite production process and polymerization process. Since Eosin-Y has benzene and pyran rings as well as a carboxyl group, it can be carcinogenic when used extensively in sensitizing industries, biomedical research labs and for diagnostic purposes. Due to its toxicity, Eosin-Y and Orange (II) create problems with the environment by being directly discharged into waste water^1–3^. The synthetic dyes known as azo dyes are difficult to break down and the byproducts are exceedingly poisonous. Dye-based products and the textile sector are dangerous sources of air pollution. These include one or more azo bonds (–N=N–) acting as chromophore groups in conjunction with aromatic structures that have functional groups like –OH and –SO_3_H^4^. Additionally, it is one of the most contaminated synthetic dyes. Wu et al. carried out synthesis of iron (III)-based metal–organic framework/graphene oxide composites with increased photocatalytic performance for dye degradation. Xuan et al.^5^ suggested that Fe_3_O_4_@Polyaniline@Au Nanocomposites exhibited excellent catalytic properties as the demonstration on the reduction of Rhodamine B dye with NaBH_4_ in comparison to the Fe_3_O_4_@SiO_2_@Au nanocatalyst. Anis et al.^6^ suggested ZrO_2_-Au nano catalyst as an excellent catalyst for degradation of organic dyes such as methylene blue and methylene orange.

Due to the interesting features, graphene has long been regarded as a wonder material and is currently the subject of intense interest. Recent developments in graphene oxide showed as potential adsorbents due to its superior mechanical and physical properties^7,8^. But simple Graphene oxide (GO) materials demonstrate poor water adsorption selectivity. Additionally, they are difficult to recycle because they are too slightly stable in water. One efficient strategy to deal with this issue is to change additional chemicals on the GO surface. The synthesis and application of different GO-based nanomaterials for the removal of contaminants from the aquatic environment were covered in a number of the published studies taken into account by Lu et al.^9^ in their study. The usage of GO/ZnO composite as a reusable adsorbent for pollutant treatment has also been carried out^10^. Rajaura et al.^11^ showed excellent antibacterial activities of reduced graphene oxide–zinc oxide nanocomposite. Xie et al.^12^ developed antibacterial activities of bacterial cellulose/graphene oxide-CuO nanocomposite films.

There are numerous researchers that are constantly developing new catalysts for the degradation of dyes, but there is a lack of information on how to remove and purify water using such catalysts and adsorbents. Similarly lack of antibacterial activity can reduce the usefulness of the material in biomedical applications. It is thus feasible to achieve required absorptivity in the present work by combining the benefit of graphene oxide composites (GOC) with SFC particles in order to produce prospective noble and new catalysts. The low cost, low toxicity and sustainability are additional benefits of **SF@GOC**. The development of supportable, recyclable materials based on graphene oxide is the main focus for the catalysis and antimicrobial activity. However, the key issues are the lengthy synthesis process, high cost and poor adsorption efficiency.

In this work, the main aim was to synthesize a nanocomposite material **SF@GOC** with its capacity to accept large-volume loadings and the necessary physical and chemical properties of the material. The modified green synthesis method was applied for synthesis of composite material. By using XRD, TEM, SEM–EDX, AFM, TEM, FTIR, N_2_ adsorption–desorption and other techniques, the structure and morphology of **SF@GOC** were systematically studied. Recyclability of the catalyst made the composite material as the future maintainable catalyst for water purification technology. This environmentally friendly catalyst can be used to clear water contaminated by organic dyes and can also be put to use in brand-new multipurpose applications. The antibacterial and antifungal activities make the synthesized material as a novel hybrid nanocomposite material that can be used in medicinal applications.

## Material and methods

### Materials

Reagents include graphite powder (Alpha Chemika, India), sulphuric acid (Loba Chemie Pvt. Ltd., Mumbai, 98%), potassium nitrate (Loba Chemie Pvt. Ltd., Mumbai, 99%), potassium permanganate (Loba Chemie Pvt. Ltd., Mumbai, 99%), hydrogen peroxide (Loba Chemie Pvt. Ltd., Mumbai, India), HCl (Molychem, India), Orange (II) (Alfa Aesa, UK), Eosin Y (Dae-Jung Chemicals & Metals Co. Ltd., Korea), sodium borohydride (Sigma Aldrich, USA) and H_2_O_2_ (Loba Chemie, India). The stock solution was made in double distilled water. Luria bertani agar (Himedia, India) used for the antibacterial assay.

### Characterization of the material

By using a powder X-ray diffraction (PXRD) from Phillips X'pert MPD diffractometer in Almelo, the Netherlands, the produced materials were examined for their two-dimensional crystalline structure over the range (10–80). By creating KBr pellets, the Fourier transform infrared (FT-IR, Perkin-Elmer Spectrometer, Massachusetts, USA) was performed. Inductively coupled plasma optical emission spectroscopy was used to identify the metals that entered the hybrid composite catalysts (ICP–OES, JY Ultima 2CHR). N_2_ adsorption–desorption data acquired at 77 K using a volumetric adsorption setup completed the BET surface area calculation (Micromeritics ASAP-2010, USA). Using the Barret-Joyner-Halenda (BJH) model, the pore width of the samples was calculated using the nitrogen adsorption isotherm's desorption branch (Supporting information). The thermal measurements and microstructural evaluation of these samples were examined by thermo-gravimetric analysis (TGA, SDT600, TA instrument, USA) temperature range of 30–800 °C with a heating rate of 10 °C min^−1^ in an air atmosphere with a flow rate of 60 cm^3^ min^−1^ using platinum crucibles. With the aid of scanning electron microscopy-energy dispersive X-ray analysis (SEM–EDX, LEO-1430, VP, UK), transmission electron microscopy (TEM, JEM 2011, Jeol Corporation, Japan) (Supporting information) and atomic force microscopy (AFM, NT-MDT, Ntegra Aura, Netherlands) analysis, the surface morphology, elemental identification, and quantitative compositional information of the material were ascertained. For the experiments, the materials were crushed finely. Dispersed in ethanol. The suspension of the material was dripped onto carbon grid and freely air dried. The measurements for AFM were performed in contact and semi contact mode on samples attached to a surface-treated mica plate with polylysine.

### Manufacturing of graphene oxide

**Figure 1:**
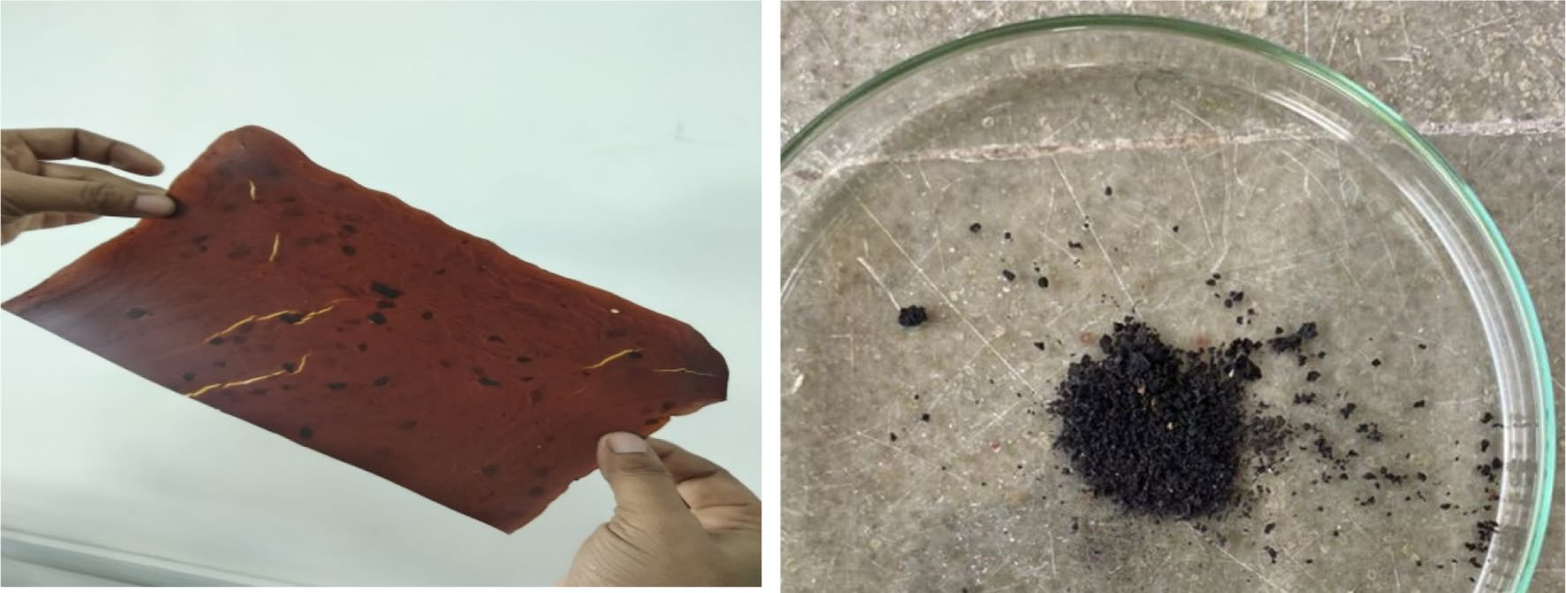
Graphene oxide (GO) sheet and strontium ferrite and graphene oxide composite (**SF@GOC**).

Hummer's method was modified in order to synthesize graphene oxide (GO)^13^. Using our experimentally ideal conditions, we altered the process to produce a unique graphene oxide material. The GO is produced using physical and chemical exfoliation methods. 2 g of graphene powder, 2 g of potassium nitrate, and 100 ml of concentrated H_2_SO_4_ (98% w/w) were introduced while magnetic stirring was going on to a 1000 ml round-bottomed flask in an ice bath (0–5 °C). After around 10 min, potassium permanganate (12 g) was progressively added to this solution. The solution was then added to a round-bottomed flask and stirred for a further hour in a water bath (35 °C). This resulted in a thick, dark paste. After adding 400 ml of distilled water and stirring the mixture for 30 min at 90 °C, 30% H_2_O_2_ was added dropwise to the mixture. The dark solution was then filtered, and the pH was then well-adjusted by rinsing it with purified water. After filtering, the cakes were gently suspended in water for an hour using a moderate Sonicator, and then centrifuged at 4000 RPM (10 min twice). Small bits of GO and other water-soluble byproducts were removed from the liquid, and the remaining material was air dried to create GO films (Fig. 1). Yield obtained was 10 g.

### Synthesis of strontium ferrite and graphene oxide composite (SF@GOC)

During synthesis of Strontium ferrite and graphene oxide composite, GO (0.9 g) was dissolved in 80 mL of ethylene glycol being ultrasonically for 6–7 h. 1.72 g of Fe (NO_3_)_3_·9H_2_O, 0.95 g of Sr(NO_3_)_2_·6H_2_O and 1.5 g of NaOH were dissolved in graphene oxide ethylene glycol solution at room temperature. The solution was set into a 100 mL Teflon-lined stainless-steel autoclave after stirring for 60 min in magnetic stirrer. The autoclave was kept at 200 °C for 6 h and then allowed to cool down to room temperature. The black precipitate was dried at 100 °C in a vacuum oven after being rinsed with EtOH a few times (Fig. 1). Yield obtained: 8.5 g.

### Catalytic reaction of Orange (II) and Eosin Y

Orange (II) and Eosin Y were catalytically degraded in a glass reactor that was connected to a condenser. Eosin Y (100 mL, 2.7 × 10^–4^ mol L^−1^), NaBH_4_ (5.4 × 10^–4^ mol L^−1^), and the catalyst **SF@GOC** (5 g L^−1^) were used to perform the reduction reaction of Eosin Y under standard circumstances. At room temperature (25 °C) and normal pressure, the reaction mixture was agitated at a rate of 180 revolutions per minute for 50 min. Using 2 g L^−1^ **SF@GOC** catalyst, the oxidation reaction of Orange (II) was conducted in 50 mL (2.7 × 10^–4^ mol L^−1^) and 50 mL H_2_O_2_ (10^–4^ mol L^−1^) for 140 min at 30 °C (room temperature), normal pressure, and 180 rpm rate. Centrifugation was used to isolate the catalyst, **SF@GOC**, after the reaction was finished. Using UV–visible spectroscopy, total degradation of Orange (II) and Eosin Y were observed (Varian, Cary-4000, USA, at maximum adsorption wavelength of 486 nm and 514 nm).

For product identification, GC–MS was employed. Following the completion of each reaction run, the products were extracted using equal parts of chloroform and the product mixture. Layers of the solution were allowed to settle after being sonicated for a while. GC–MS analysis was performed on 0.6 μL of the mixture’s extracted layer (Shimadzu GC 2010, USA). The injection temperature in GC–MS was 250 °C. The initial temperature was held at 50 °C for 5 min before being increased to 180 °C at a rate of 10 °C min^−1^, maintained at this temperature for 5 min, and then increased to 250 °C at the same rate, again maintained for 5 min.

### Antimicrobial activity

Antibacterial activity of the GO and **SF@GO** composite was detected using the disc diffusion method. Briefly, the luria bertani agar (Himedia, India) used for the antibacterial assay. The four bacterial cultures are *Pseudomonas aeruginosa (P. aeruginosa)*, *Escherichia coli* (*E. coli*), *Bacillus subtilis* (*B. subtilis*) and *Staphylococcus aureus* (*S. aureus).* The loopful of bacterial culture was incubated in the 50 mL of LB broth and incubated in shaking conditions at 37 °C overnight. The growth was measured spectrophotometrically at 600 nm and once it reached to exponential phase (OD at 0.5) and the rate of CFU/mL is about 2.7 × 10^8^ CFU/mL then the culture was taken out from the incubation to avoid further growth. From the activated culture, 1 mL of suspension was added on Luria bertani agar medium. The small films were dipped in the GO and **SF@GOC** solution prepared n polyethylene glycol. The films were kept in medium to absorb the solution and further the same film was used for the antibacterial activity. The zone of inhibition was Verniercaliper^14^. The experiment was repeated three times against each strain and the average values were recorded.

### Growth curve analysis

The interaction of GO and **SF@GOC** on the bacterial growth was analyzed by performing turbidometric assay. Briefly, the bacterial suspension (containing 1 × 10^6^ to 1 × 10^7^ CFU/mL) was used as inoculum as starting the growth curve measurement. The three concentrations of 2, 5 and 10 mg/mL were taken for studying growth of microbes in response to ethylene glycol (EG), graphene oxide (GO) and **SF@GOC**. The absorbance of the samples was analyzed at 600 nm using UV visible spectrophotometer (Shimadzu 1800, Japan). The sample readings were taken till 24 h with interval of 2, 4, 6, 8, 10, 12, 18 and 24 h of incubation at 37 °C and 150 rpm in shaking condition in incubator. All treatments were performed in triplicates.

### Antimicrobial mode of action

Mode of inhibition against the microbes was studied using scanning electron microscopy (SEM). Briefly, SEM was carried out for observation of morphological changes in the bacterial culture after exposure with GO and **SF@GOC**. Microbial cultures were activated by inoculating the bacterial strains overnight at 37 °C at 130 rpm in shaking conditions. The 500 µL blank broth and culture treated with GO and **SF@GOC** was collected after 24 h of growth. The collected biomass was fixed overnight with 3% glutaraldehyde. Further, the cells were fixed by dehydration process using a gradient series of ethanol with incubation time of 10 min and final step using 100% ethanol for 15 min. The dehydrated cells were spotted on glass surface and kept for ethanol evaporation at room temperature. The dried samples were gold coated for SEM imaging using JEOL JSM 6301F (Carl Zeiss, Germany).

### Antifungal assay

For determination of the antifungal activity, the GO and **SF@GOC** activity against the *C. albicans* and *C. tropicalis* were carried out by broth microdilution method. Briefly, the fungal cultures with concentration of 105 CFU/mL were dispersed in the 50 mL of the nutrient broth. This broth was further added in the 96 well microtiter plates. The Final concentration of GO and **SF@GOC** was kept 0.25, 0.5, 1, 2, 5 and 10 mg/mL. The uninoculated broth served as the control for the experiment. The microtiter plate was incubated at 37 °C for 24 h. The optical density was measured at 570 nm using the ELISA reader (EliQuant, Meril Diagnostics, India).

## Results and discussion

### Physico-chemical characterization

#### Powder X-ray diffraction analysis (PXRD)

**Figure 2:**
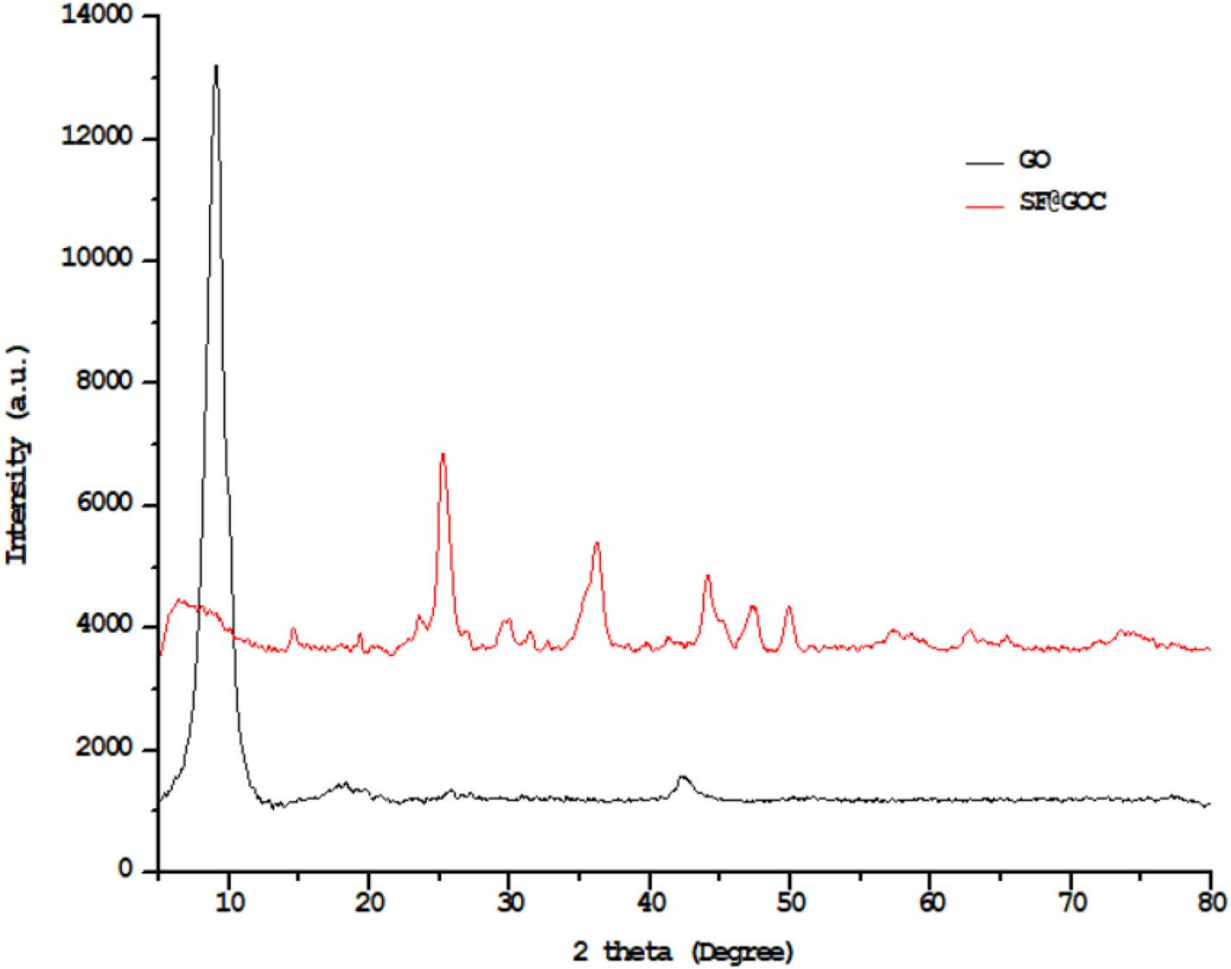
High angle powder XRD of GO and **SF@GOC.**

Powder X-ray diffraction was employed to characterize and evaluate the crystalline structure of the synthetic material. Figure 2 demonstrated the XRD patterns of GO and **SF@GOC**. All detectable peaks in wide angle PXRD were indexed. In the high angle 2 theta range of 5° to 80°, the patterns were seen. The sample showed 001 reflection at roughly 10°, which corresponds to a d_001_ = 8.80 basal spacing. The interlayer gap and distinct peak at 2 theta value of 10.0° show that oxygen-containing functional groups (–OH, –COOH) were present during the oxidation of graphite. The semi-crystalline and amorphous features of the sample were shown by small peaks in the XRD pattern at various positions at 2 theta values 25.2° and 42.7° with 4.001 and 2.128 interlayer spacing, respectively^15^. In **SF@GOC**, a clear peak could be seen at 25.6°, which corresponded to d spacing 3.47. The interplanar spacing (d_hkl_-value) for the peak placed at 29.8°, 35.0°, 42.6°, 43.9°, 49.7° and 56.7° are 2.99, 2.55, 2.11, 2.06, 1.83 and 1.62 nm corresponding to the planes (220), (311), (222), (400), (422), and (511). This shows that strontium ferrite is efficiently deposited on the surface of the GO, generating a well-structured composite material **SF@GOC**. According to the ICP-OES study, the loading amounts achieved for iron and strontium are 9.7% and 18.2%, respectively. This result confirmed the achievement of the **SF@GO** composite material^16^.

#### Microscopic characterization (scanning electron microscopy-energy dispersive X-ray analysis (SEM–EDX) and atomic force microscopy (AFM) analysis

**Figure 3:**
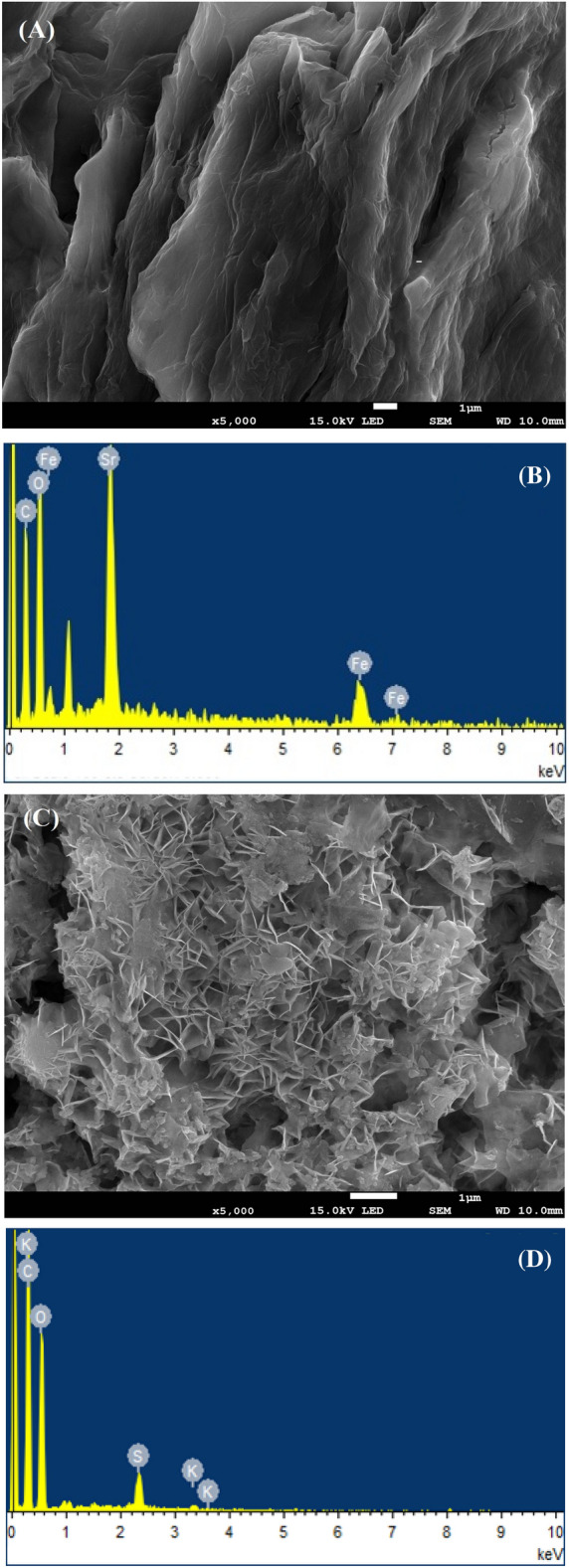
SEM images of GO (**A**), EDX of GO (**B**), SEM of **SF@GOC** (**C**), EDX of **SF@GOC** (**D**).

More creases, folds and agglomerations were visible in SEM micrographs (Fig. 3A) and the edges of the sample had a two-dimensional sheet-like structure. Insertion of oxygen-containing functional groups caused the observed multiple lamellar layer structure and thickness at the edge, which was also reported in the literature^16^. EDS spectra was employed to establish elemental mapping of GO (Fig. 3B). In the **SF@GOC** SEM picture, which depicted a powdered material with a significant irregular spindle-shape structure has been shown in Fig. 3C. It has been demonstrated that monodispersed strontium ferrite composites can spontaneously adhere to GO sheet's two-dimensional structure. The micrograph showed that strontium iron composite particles had been effectively used to enhance GO flexible sheets. This established that the particles were dispersed all over the GO giving collapsed nano stacks. Because of hydrogen bonding and Van der Waal forces, composites may have formed as GO and self-assembled^17^. EDS spectra for **SF@GOC** has been given in Fig. 3D. Using Table 1, a percentage of the elements (weight %) that are present in both GO and **SF@GOC** has been provided. The textural characteristics of GO, which exhibited a distinctive lamellar shape, were confirmed and demonstrated by TEM micrographs (Supporting information, Fig. S1A–D).

**Table 1:** Elemental analysis from SEM–EDX.

Graphene oxide (GO)			
Carbon (C)	Oxygen (O)	Sulphur (S)	Potassium (K)
56.8%	40.4%	2.41%	0.43%

Strontium ferrite and graphene oxide composite (**SF@GOC**)			
Carbon (C)	Oxygen (O)	Iron (Fe)	Strontium (Sr)
36.7%	35.5%	9.68%	18.2%

**Figure 4:**
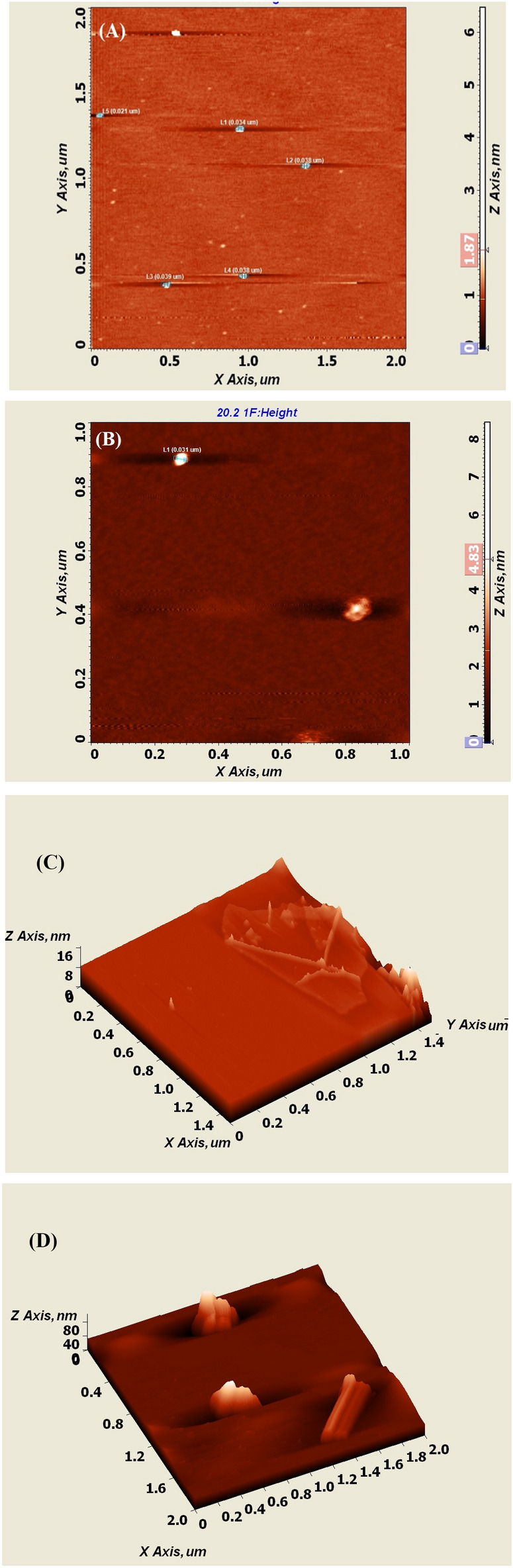
AFM images of GO and **SF@GOC** with particle size (**A**,**B**); images with corresponding 3 D views (GO: **C** and **SF@GOC**: **D**).

AFM results have been used to display the composites with the various surface morphologies of GO and **SF@GOC.** In our analysis, AFM demonstrated and validated the expected values for hydrated single-layer GO, which ranged in lateral dimension from 0.021 to 0.039 µm (Fig. 4A). Again, **SF@GOC** has been measured at 0.031 µm (Fig. 4B). Figure 4C,D, respectively, depict GO and **SF@GOC** for clear topography scan with 3D views^18^. In **SF@GOC**, the immobilization-induced structural alterations are again quite clear. The modifications may result from the loss of oxygenated functional groups. The roughness histogram of the material from the AFM has been included (Supporting information, Fig. S7).

#### Thermogravimetric analysis (TGA)

**Figure 5:**
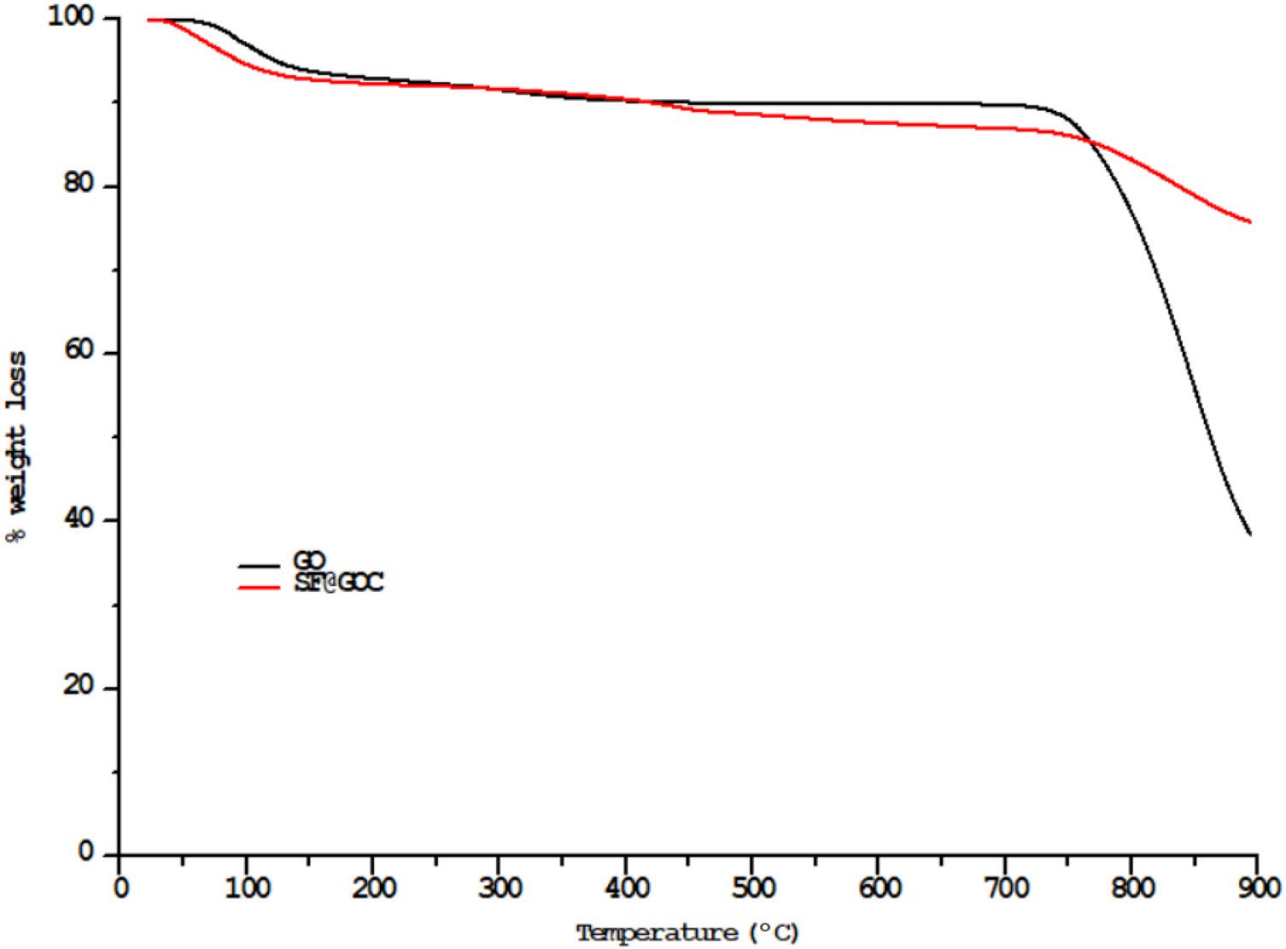
TGA curves of GO and ***SF@GOC****.*

From 21 to 900 °C, TGA measured minute weight losses of about 3.9 and 7.1% for GO and **SF@GOC**, respectively. The release of water vapours might be the main cause of this weight loss. Carbonization resulted in consistent weight losses of GO and **SF@GOC** between 28 and 900 °C. Figure 5 shows the weight percentage loss of the composites as a plot. The outcomes validated that the based on their breakdown temperatures, the primary mass loss of GO and **SF@GOC** can be explained: 100 °C for water elimination, gradual mass loss between 150 and 900 °C for the oxidative pyrolysis of carbon framework of graphene oxide. The thermal stability of our composite materials is compared with similar literature reports as well^19^. The BET analysis was used to calculate the surface area of the GO and **SF@GOC**. N_2_ adsorption–desorption isotherm type IV was used to characterise the BET analysis for the produced composite (Supporting information, Fig. S2 and Table S1).

### Catalytic reactions of Orange (II) and Eosin Y

#### Blank experiments

Initially the effectiveness of the catalysts for the catalytic oxidation of Orange (II) and reduction of Eosin Y, a set of blank experiments were carried out under the reaction conditions: (i) Orange (II) (2.7 × 10^−4^ mol L^−1^) without a catalyst and H_2_O_2_, (ii) Orange (II) (2.7 × 10^−4^ mol L^−1^) and H_2_O_2_ (1:1 molar ratio) without a catalyst, (iii) Orange (II) (2.7 × 10^−4^ mol L^−1^) with **SF@GOC** as the catalyst (2 g L^−1^), and (iv) Orange (II) (2.7 × 10^−4^ mol L^−1^) and H_2_O_2_ (1:1 molar ratio) with **SF@GOC** as the catalyst (2 g L^−1^). Similarly for Eosin Y, i) Eosin Y (2.7 × 10^−4^ mol L^−1^) without a catalyst and NaBH_4_, (ii) Eosin Y (2.7 × 10^−4^ mol L^−1^) and NaBH_4_ (5.4 × 10^−4^ mol L^−1^, 1:1 molar ratio) without a catalyst, (iii) Eosin Y (2.7 × 10^−4^ mol L^−1^) with **SF@GOC** as the catalyst (5 g L^−1^), and (iv) Eosin Y (2.7 × 10^−4^ mol L^−1^) and NaBH_4_ (5.4 × 10^−4^ mol L^−1^, 1:1 molar ratio) with **SF@GOC** as the catalyst (5 g L^−1^). The reactions were carried out at 298 K, atmospheric pressure, 200 rpm stirring and a time interval of 2 h. No measurable conversion could be detected for blank experiment of both the targeted dyes.

#### Effect of the reaction time

**Figure 6:**
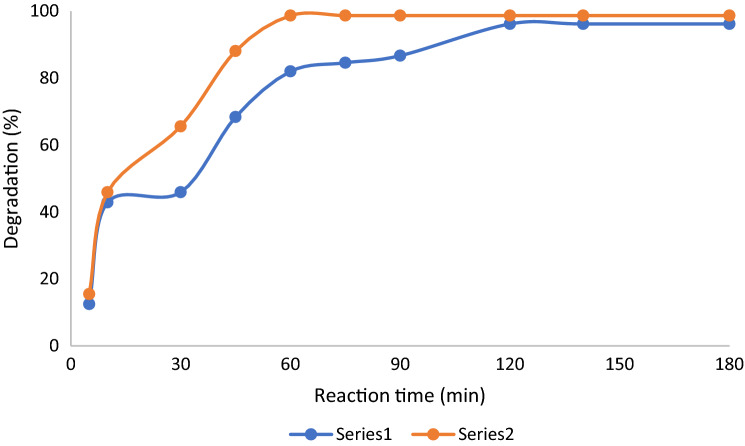
Effect of reaction time on Orange (II) (Series 1) and Eosin Y (Series 2) degradation.

**Figure 7:**
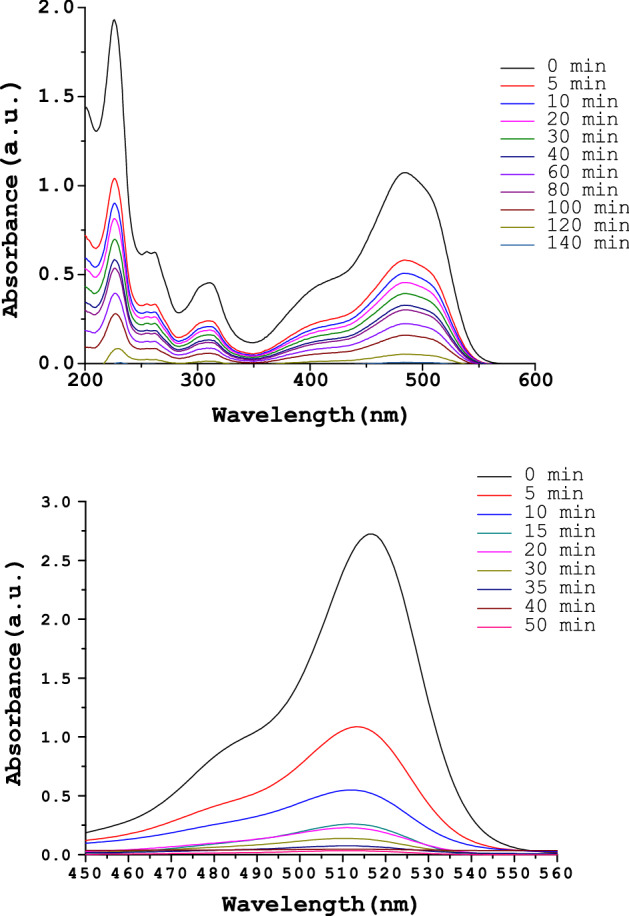
Time dependent UV–Visible spectra of Orange (II) and Eosin Y solutions with **SF@GOC*.***

An increase in the reaction time from 5 to 180 min showed improved Orange (II) degradation, as shown in Fig. 6. In a series of reactions, which were carried out in this time interval with Orange (II) (2.7 × 10^−4^ mol L^−1^) and H_2_O_2_ (10^−4^ mol L^−1^) at 298 K using **SF@GOC** as catalyst with a load of 2 g L^−1^, the conversion increased 12.5 to 96.2% respectively. Whereas reduction of Eosin Y (100 mL, 2.7 × 10^–4^ mol L^−1^), NaBH_4_ (5.4 × 10^–4^ mol L^−1^) and the catalyst **SF@GOC** (5 g L^−1^) showed that the conversion increased from 15.5 to 98.7% within 60 min when the reaction was carried out from 5 to 180 min (Fig. 6). The catalyst **SF@GOC** was found to be capable catalyst, indicating that the in-situ synthesis of metal ferrite composite might have left some metals strontium and iron on the available sites of GO, which are accountable for changing more reactants. The absorbance peaks (Orange (II): 486 nm and Eosin Y: 514 nm) became weaker in intensity with time and totally disappeared after 120 min and 60 min respectively (Fig. 7). This directs that the Orange (II) and Eosin Y were degraded totally into its mineral components. In our work, the products of the catalytic degradation of Orange (II) and Eosin Y were monitored by GC–MS analysis.

#### Effect of pH for leaching experiment

Since transition metal ions (Sr^3+^, Fe^3+^) have been present in the composite material **SF@GOC**, it was critical to determine whether they could result in secondary pollution. By aggressively mixing 0.1 g of the catalysts with 50 mL of water for 3 h at 298 K (the temperature at which the reactions were conducted), pH 3.0, 4.0, 5.0, 6.0, 7.0, 8.0, and 9.0, it was possible to study the leaching of the metals from the catalysts. The mixture was centrifuged to determine the concentrations of Sr and Fe in the aqueous layer. From **SF@GOC**, adsorbent was released in two different ranges: 0.25–0.99% (Fe) and 0.01 to 0.12% (Sr). Although the leaching is significantly influenced by the pH of the standard, it was discovered that excessive release occurred in basic medium. Overall, the leaching was minimal and has no impact on the concentration of metals in water, which was greater than the allowable limits even when drinking water quality was taken into account. According to the leachability experiment, the amount of Sr and Fe released into the water would result in a concentration that is far lower than the value recommended by the World Health Organization (WHO) guideline (for iron in natural fresh water 0.3 mg L^−1^ and for strontium in drinking water 7 mg L^−1^). Therefore, use of catalyst will result in no secondary water pollution.

#### Product analysis

The identification of reaction products from the catalytic oxidation of Orange (II) and reduction of Eosin Y was carried out with **SF@GOC** by means of the GC–MS method. The mechanism is based on **·**OH radicals. These radicals have their origin either in metal oxides formed during calcination of the catalysts or in the breaking of hydrogen peroxide (or water) molecules under the influence of the transition metal cations. The formation of **·**OH radicals on the surface of the catalysts will follow from interactions between the excited O-atoms of the catalyst and H-atoms cleaved from the substrate or even water because the reactions were carried out in an aqueous solution. The participation of dissolved oxygen in **·**OH radical formation is unlikely because this will result in a drastic reduction in oxidation with increasing reaction temperature (consequent to a decrease in dissolved oxygen level). The breakdown of Eosin Y employing **SF@GOC** as a catalyst shows that the carboxyl group-containing benzene and pyran rings of Eosin Y are destroyed and broken down into simpler acid components, which will subsequently degrade into their mineral components. The products, including 2-(2-formylphenyl)-2-carboxylate, and (1Z,4E)-1,5-dibromo-3((Z)-2carboxyvinyl)-6-oxohexa-1,4-dien-2-olate could be identified by GC–MS. Possible catalytic degradation of Eosin Y mechanism was proposed based on degradation products can be depicted in Figs. S3 and S4 (supplementary information). Based on the products detected, it is assumed that the degradation process is initiated by cleavage of the C−N bond due to the oxidative attack of hydroxyl radicals which then undergoes ring-opening reactions leading to the formation of short-chain aliphatic acids. Finally, these organics are mineralized into CO_2_ and H_2_O. GC–MS could identify 1,2-benzene dicarboxylic acid, phthalic anhydride as the products of orange II degradation. Mechanism has been given in Fig. S5 (supplementary information).

### Antimicrobial activity

**Figure 8:**
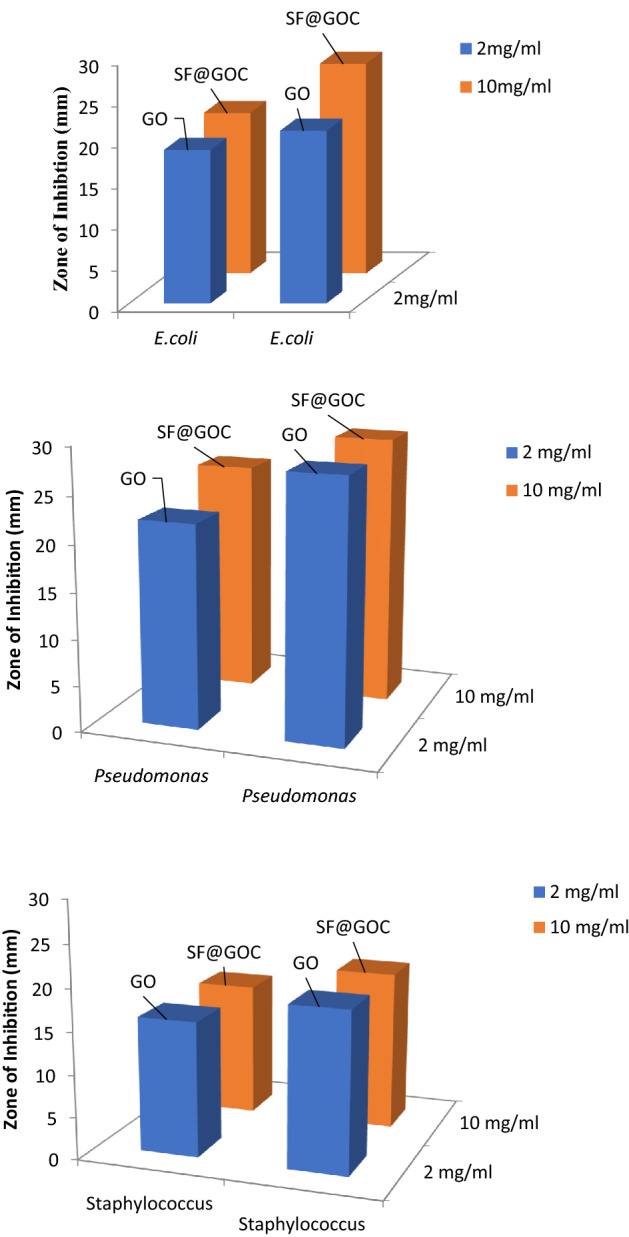
Zone of inhibition against *Escherichia coli, Pseudomonas aeruginosa,* and *Staphylococcus aureus* species.

In the present study, all the strains except the *bacillus* species were found to be sensitive to GO and **SF@GOC**. The zone of inhibition was 6 mm for the *Pseudomonas aeruginosa* species, 3–4 mm for *Escherichia coli and Staphylococcus aureus* in 2 mg/mL and 10 mg/mL concentration (Fig. 8). There was no inhibition observed in *Bacillus* species with synthesized GO and **SF@GOC** compound. This can be due to the complicated peptidoglycan arrangement features which can be related correlated with the factors associated with the synthesis of the peptidoglycan The AFM studies indicate that synthesized the GO has edges which can perforate the bacterial outer membrane and molecule can easily get inside and inhibit the bacterial strains. However, it also depends on the peptidoglycan layer in the bacteria^20,21^. Additionally, these sharp edges can break the hydrophobic bonds and as a result can phospholipids are extracted^22^. This cell membrane disintegrity leads to stoppage of vital functions like respiration, membrane exchange mechanisms, energy transmissions etc^23,24^. There are also other factors associated with the inhibition mechanism by GO and **SF@GOC** material. Possible mechanisms involve the outer layer is covered with the GO and **SF@GOC** which leads to possible lower nutrient reflux and due to that the bacteria come to arrest stage and finally to the death phase. The other possible mechanism can be the bacterial entrapment in the GO and **SF@GOC** synthesised material which can lead to no respiration and membrane transport. Also, the size of synthesised material usually affects the bacterial inhibition. Based on spectrophotometric and AFM data the size of GO is 0.039 µm and **SF@GOC** is 0.031 µm. This size is resembling to the bacterial size used in the study. From this, it can be also assumed that possible mechanism of inhibition can be entrapment, cutting due to material edges or possible covering of material to the bacterial membrane, although the exact mechanisms need further careful observation and in-depth studies for better understanding of inhibitory mechanism and exact conclusion. The A and B zone of inhibition against 2 mg/mL concentration the *Pseudomonas aeruginosa*; C and D zone of inhibition against the *E. coli* and *Staphylococcus* strain 10 mg/mL concentration can be depicted in Fig. S6 (Supporting information).

### Growth curve analysis

**Figure 9:**
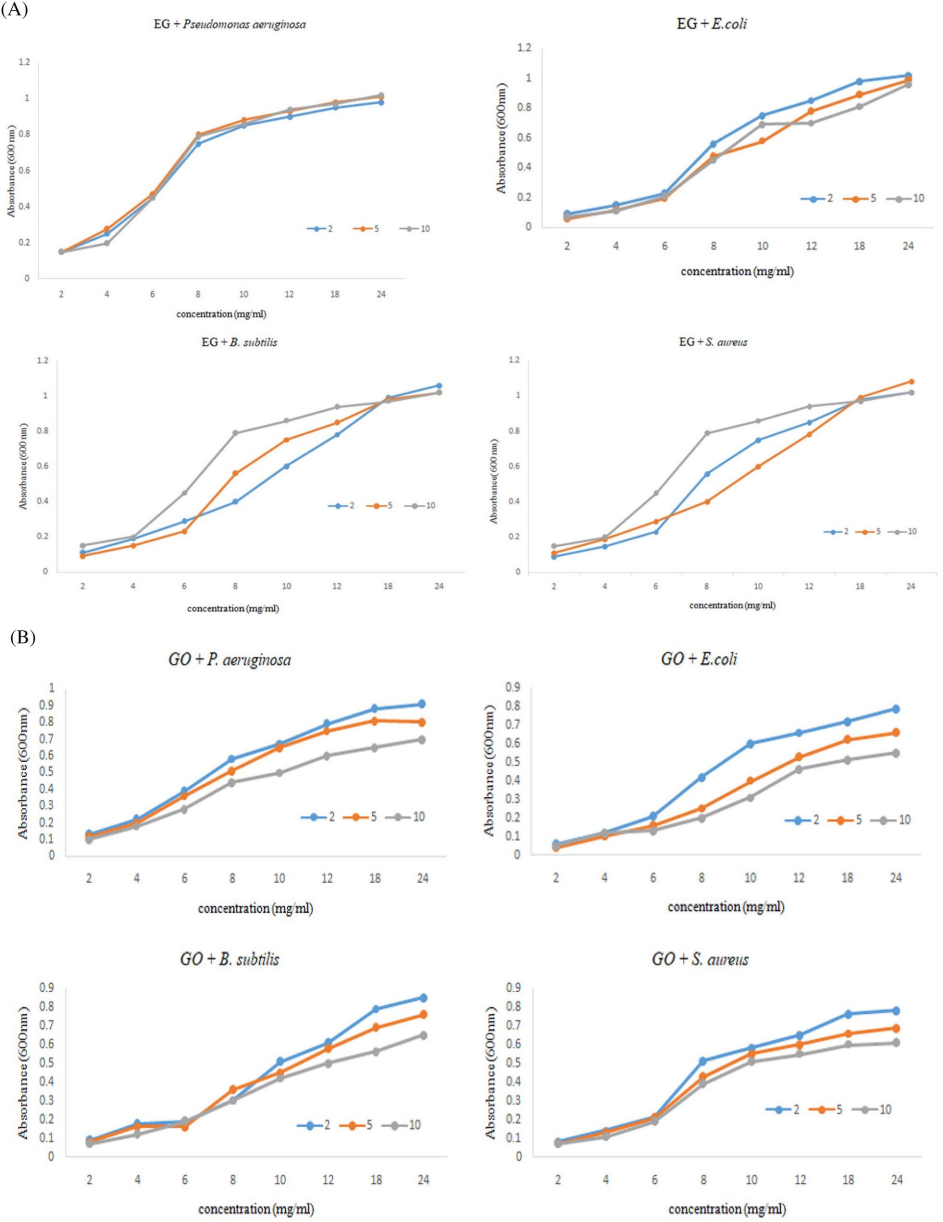

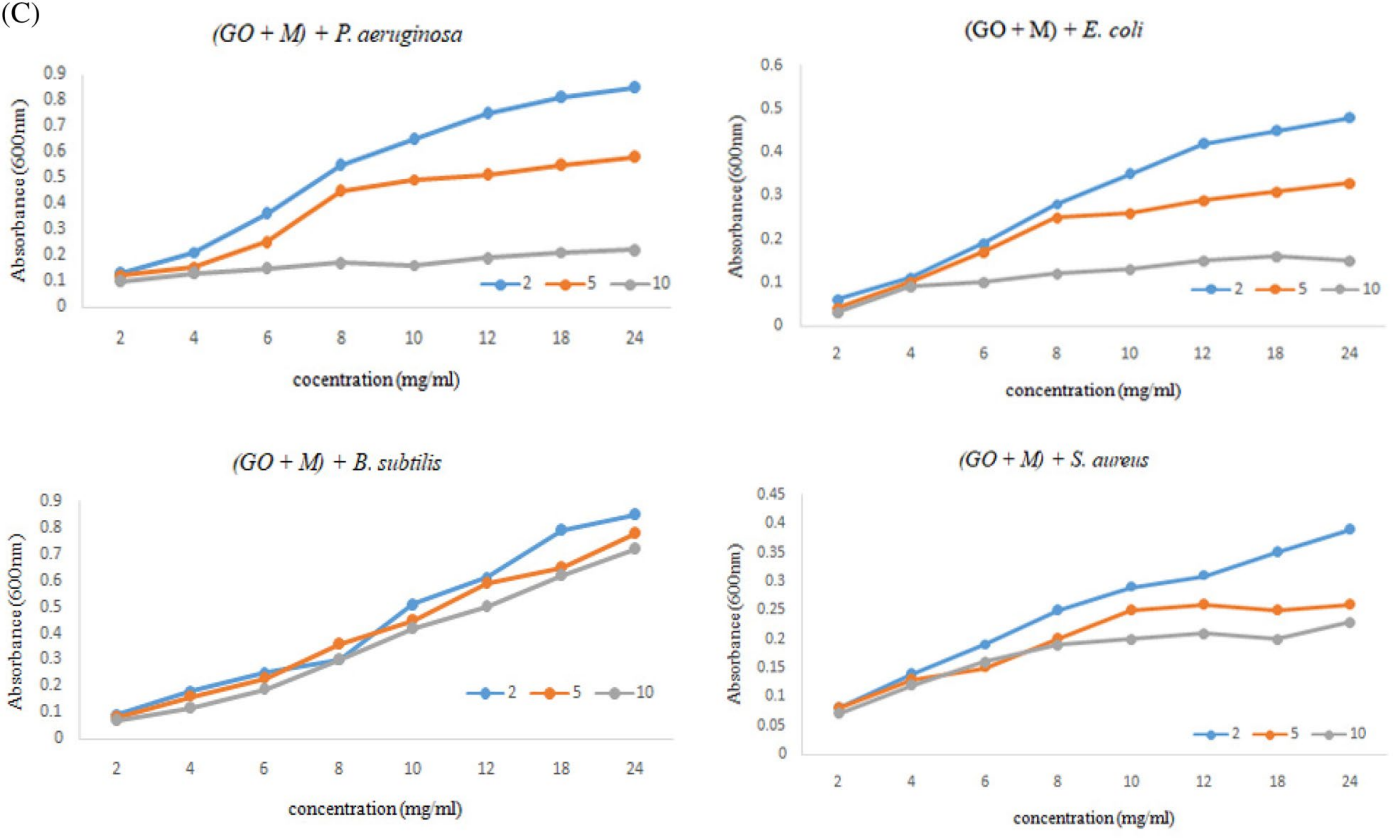
Microbial growth curve analysis. For EG: Growth curve of the *P. aeruginosa*, *E.coli*, *B. subtilis* and *S. aureus* exposed to the different concentrations (**A**). For GO: Growth curve of the *P. aeruginosa*, *E. coli*, *B. subtilis* and *S. aureus* exposed to GO with different concentrations (**B**). For **SF@GOC (GO + M)**: Growth curve of the *P. aeruginosa*, *E. coli*, *B. subtilis* and *S. aureus* exposed to **SF@GOC** with the different concentrations (**C**).

Microbial growth curve analysis represents interesting way to study antimicrobial activity as assay reflect two important parameters to be studied, the concentration of graphene composite material and the exposure time. In the present study the **SF@GOC** showed better inhibition to all the bacterial strain tested with time frame of 24 h. However, among the strains used, the better inhibition was observed in the *S. aureus* and *E. coli* strain as compared to *P. aeurginosa* strain at 10 mg/mL of concentration. Similar results were published where graphene oxide based nanomaterial showed significant inhibition activity against *E. coli*^24^*.* The growth curve analysis result supports the antimicrobial results where no significant inhibition was observed in *B. subtilis* strain using GO or **SF@GOC**. This observation can be correlated with the previously published reports suggesting that GO does not inhibit the microorganism growth as the growth of microbes exceeded in terms of absorbance with that of the nanomaterial which had not been in complete contact with the microbes^25,26^. As per the literature, microbial inhibition depends on the size, structure, layers, purity and internal arrangement there are diverse sets of results and its correlation with the characteristics of GO based nanomaterials^25–27^. Additionally, reports also suggest that the small surface coated GO nanomaterials show better microbial inhibition compared to the nanocomposite materials possessing larger sheets^28^. (Fig. 9).

### Antimicrobial mode of action

**Figure 10:**
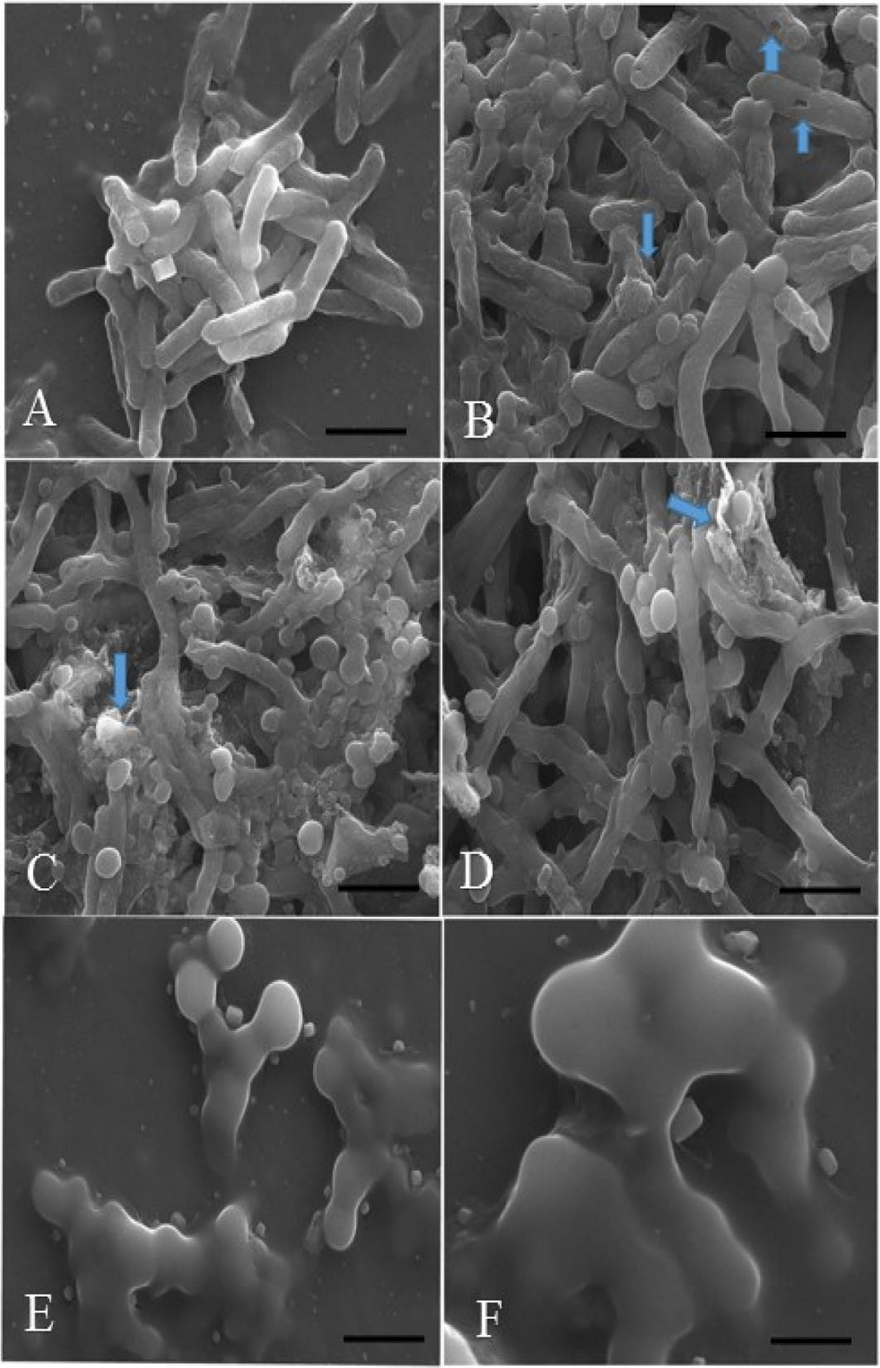
Antimicrobial mode of action. (**A**) GO exposed *E. coli* cells. The cells were found to be coated with the graphene oxide sheet. (**B**) **SF@GOC** exposed *E. coli* cells. The cells were found to be wrapped with the **SF@GOC** sheets and also the perforation/puncture in the cells are observed (marked with the blue arrows). (**C**) **SF@GOC** exposed *P. aeruginosa* cells. The cells were enclosed in the nanomaterial. (**D**) **SF@GOC** exposed *P. aeruginosa* cells. The cells leakage was observed after complete wrapping of the cells. (**E**,**F**) **SF@GOC** exposed *S. aureus* cells. Complete shape deformation observed. *(scale bar of all images are 1 μm).

In present study, for studying the mode of antimicrobial mechanism, SEM technique was utilized to observe the extent of cell damage and morphological changes in bacteria using the GO and **SF@GOC** material. The results for the inhibition using GO and **SF@GOC** on *E. coli, P. aeruginosa* and *S. aureus* is depicted in Fig. 10. In case of the *E. coli*, the GO makes a thin layer on the outer membrane of the cells and **SF@GOC** punctures the membrane and leaks the cell constituents (Fig. 10A, B). Our findings support the hypothesis that graphene-based nanomaterial exhibit strong antimicrobial action by coming in direct contact with the bacterial cell membrane^27,29,30^. This rupture/perforation can be correlated with the mechanical puncture leading to cell death^31^. Interaction of the **SF@GOC** on the *P. aeruginosa* was also found to be similar in nature as that of *E. coli* where the sheet formation was observed higher compared to the *E. coli*. In case of *P. aeruginosa*, the nanomaterials seem to cause the wrapping and further leakage of cytoplasmic fluids (Fig. 10C,D). These observations can be attributed due to presence of the higher lipopolysaccharides in gram negative bacteria^31^. The antibacterial activity of **SF@GOC** was uniquely different in case of *S. aureus*. The cell membrane was initially been disintegrated further leading to the deformation of cells. Our findings were similar to those previous reports suggesting that the graphene-based nanomaterials display higher antibacterial activity against gram positive bacteria compared to that of gram-negative bacteria^31–33^. SEM analysis reveals that the bactericidal mechanism for **SF@GOC** is same in both gram-negative bacteria.

### Antifungal assay

**Figure 11:**
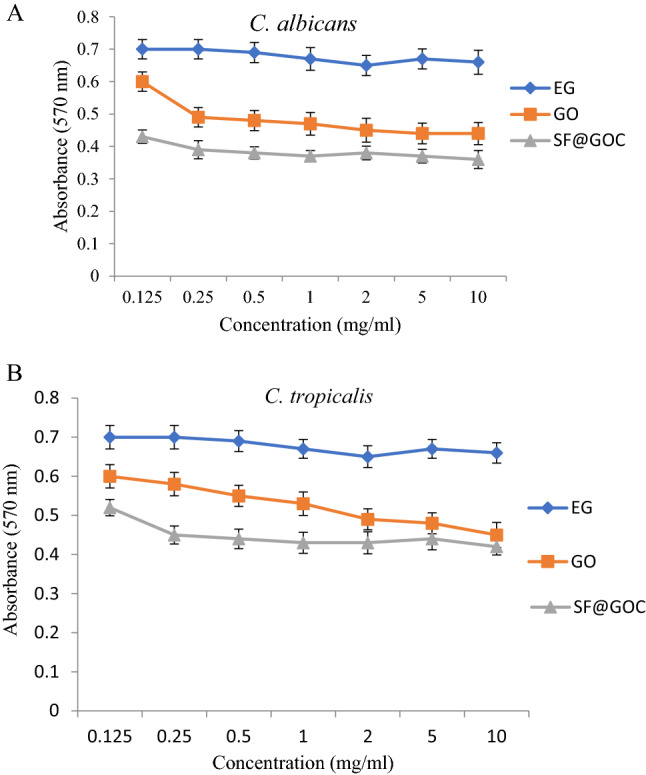
The antifungal activity test of GO and **SF@GOC** with *C. albicans* (**A**) and C. tropical (**B**) by broth microdilution method.

The antifungal activity of GO and **SF@GOC** is shown in Fig. 11. From the graphs, it is clear that antifungal activity of the GO is lower than that of **SF@GOC**. On comparison of the activity among the candida strains, the inhibition of *C. albicans* seems to be more compare to the *C. tropicalis* strain. The MIC of the C. *albicans* treated with **SF@GOC** was found to be 0.125 mg/mL and *C. tropicalis* was found to be 0.25 mg/mL. The inhibitory mechanism described is different compared to bacteria as the using yeast cell there might be discrepancy in zone of inhibition diameter^34,35^. The difference in the antifungal inhibition values might be due to competence between the synthesis part GO and **SF@GOC** which can further lead to the slower and less release. This mechanism of slow release can be correlated with the wrapped carbon structure of the **SF@GOC**. Additionally, the difference in the antifungal activity among the *candida* strains can be correlated with the movement of ions release (slower and faster) of synthesized nanoscrolls in **SF@GOC**^36^. The more it was wrapped with the carbon nanoscrolls, stronger the antifungal activity was reported.

### Recyclability of the SF@GOC after dye degradation

**Figure 12:**
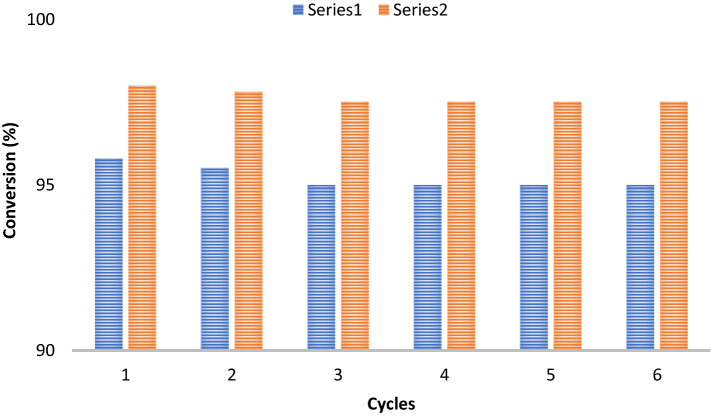
Recyclability of **SF@GOC** after catalytic reaction with Orange (II) (Series 1) and Eosin Y (Series 2).

Following the catalytic degradation of Orange (II) and Eosin Y, under optimal reaction conditions (Orange (II): 2.7 × 10^–4^ mol L^−1^, 50 mL; H_2_O_2_: 10^–4^ mol L^−1^, 50 mL; catalyst load: 2 g L^−1^; Eosin Y: 2.7 × 10^–4^ mol L^−1^, 100 mL; NaBH_4_: 5.4 × 10^–4^ mol L^−1^, 0.025 g; catalyst load: 5 g L^−1^), the objective was to assess the catalyst's resistance to degrading reactions, which is a prerequisite for its use on an industrial scale. Following the initial catalytic reaction, the nanocomposite was extensively washed with distilled water while the utilized **SF@GOC** catalyst was separated by filtration. After being dried for a whole night at 100 °C in vacuum desiccators, the resulting nanocomposite was reactivated which was then employed as a catalyst in the following catalytic reaction. The same catalyst was used for six catalytic runs (Fig. 12), and there was no noticeable performance degradation observed.

## Conclusions

This work presented a novel and low-cost material for more an effective groundwater dye removal. The efficiency of **SF@GOC** as an adsorbent for the dye degradation of groundwater and aqueous solutions has been demonstrated. As a result of its exceptional organic–inorganic hybrid physicochemical features, the recently developed strontium ferrite graphene oxide composite, **SF@GOC**, may have applications in catalysis. The environmentally friendly catalyst functions as a dynamic heterogeneous catalyst for the catalytic breakdown of Eosin Y and Orange (II), two dyes that are harmful water pollutants. In contrast to past literature findings, it was successfully done to completely degrade the dyes Eosin Y and Orange (II) into acidic chemicals, which may then be further mineralized to CO_2_ and H_2_O under mild reaction conditions. Another benefit of the catalytic degradation process is the recyclable nature of the eco-friendly catalyst. The antibacterial activity of **SF@GOC** was established by Zone of inhibition. Although the exact mechanisms require more careful observation and in-depth studies for a better understanding of the inhibitory mechanism. All the strains (*P. aeruginosa, E. coli* and *S. aureus*) except the *bacillus* species were found to be sensitive to GO and **SF@GOC**. Mode of action analysis by FESEM showed that our findings support the hypothesis that graphene-based nanomaterial exhibit strong antimicrobial action by coming in direct contact with the bacterial cell membrane. The antifungal activity of the GO is lower than that of **SF@GOC**. On comparison of the activity among the candida strains, the inhibition of *C. albicans* seems to be more compared to the *C. tropicalis* strain. The benchmark of our research group's future planning will be the conversion of the sustainable new composite materials for application in actual water purification operations, energy storage and coating material for bioelectronics.

## Supplementary Information

Supplementary Information.

## Data Availability

The data used to support the results of this study are included within the article.
