# Supplementary material for: Development of sustainable strontium ferrite graphene nanocomposite for highly effective catalysis and antimicrobial activity
Source: Sci Rep. 2023 Apr 24;13:6678. doi: 10.1038/s41598-023-33901-w (PMC10126001; doi:10.1038/s41598-023-33901-w)
Supplement: Supplementary file 1 — Supplementary Information. [file 41598_2023_33901_MOESM1_ESM.docx]

**Development of sustainable Strontium Ferrite Graphene Nanocomposite for highly effective catalysis and antimicrobial activity**

**Suranjana V. Mayani^*1^, Sandip P. Bhatt^1^, Vishal J. Mayani^2^ Gaurav Sanghvi^3^**

^*1^Department of Chemistry, Marwadi University, Rajkot-Morbi Road, P.O. Gauridad, Rajkot 360003, Gujarat, India, E mail: [suranjana.mayani@marwadieducation.edu.in](mailto:suranjana.mayani@marwadieducation.edu.in), ORCHID: 0000-0002-2624-3305

^2^Hansgold ChemDiscovery Center (HCC), Hansgold ChemDiscoveries Pvt. Ltd. Rajkot, Gujarat, India

^3^Department of Microbiology, Marwadi University, Rajkot-Morbi Road, P.O. Gauridad, Rajkot 360003, Gujarat, India

***Transmission Electron Microscopy (TEM) Analysis***

The textural characteristics of GO, which exhibits a distinctive lamellar shape, were confirmed and demonstrated by TEM micrographs, as shown in Figure S1 (A). Broad fields were visible in TEM images. Due to their single-layer structure for GO and **SF@GOC**, GO nanosheets frequently exhibit the usual bidimensional morphology and lateral dimensions in the micrometer range with light contrast. The SFC particle had been effectively incorporated into the GO sheet, according to the **SF@GOC** composite (Figure S1 B). We can assume that a hydrogen bond between the functional groups OH and COOH on the surface of GO sheet and SFC is the primary cause of this recombination (Kuang et al., 2016). The metal ferrite composite was distributed evenly across the surface of the GO, and the black aggregates were well-decorated, according to the TEM images at various magnifications (Figure S1 (C, D)).

| 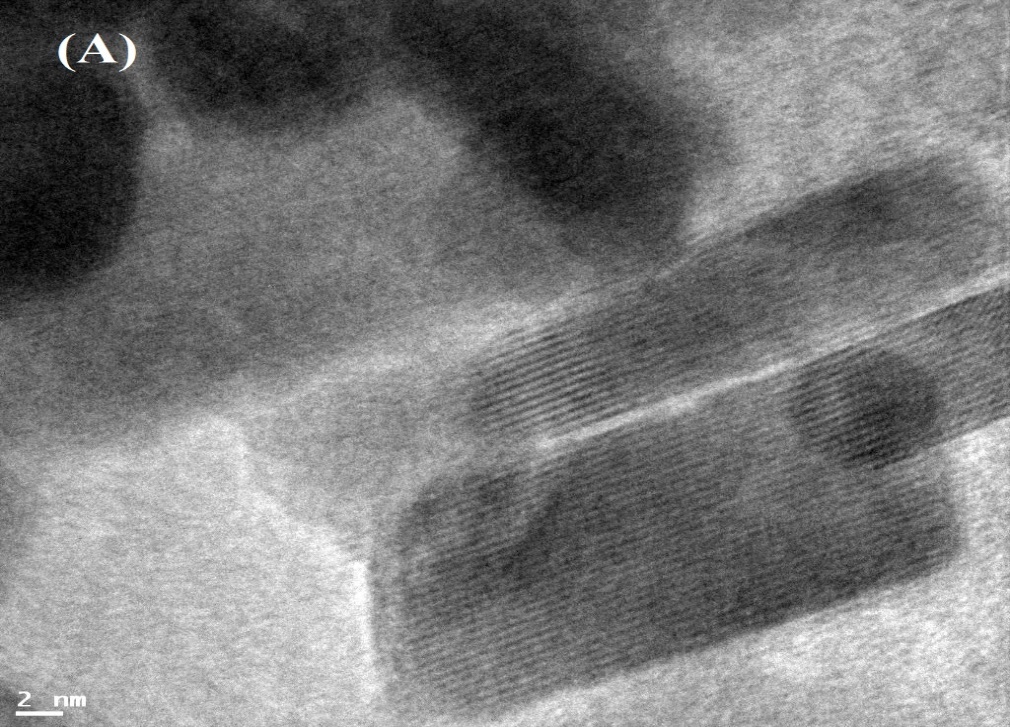 |
| --- |
|  |
| 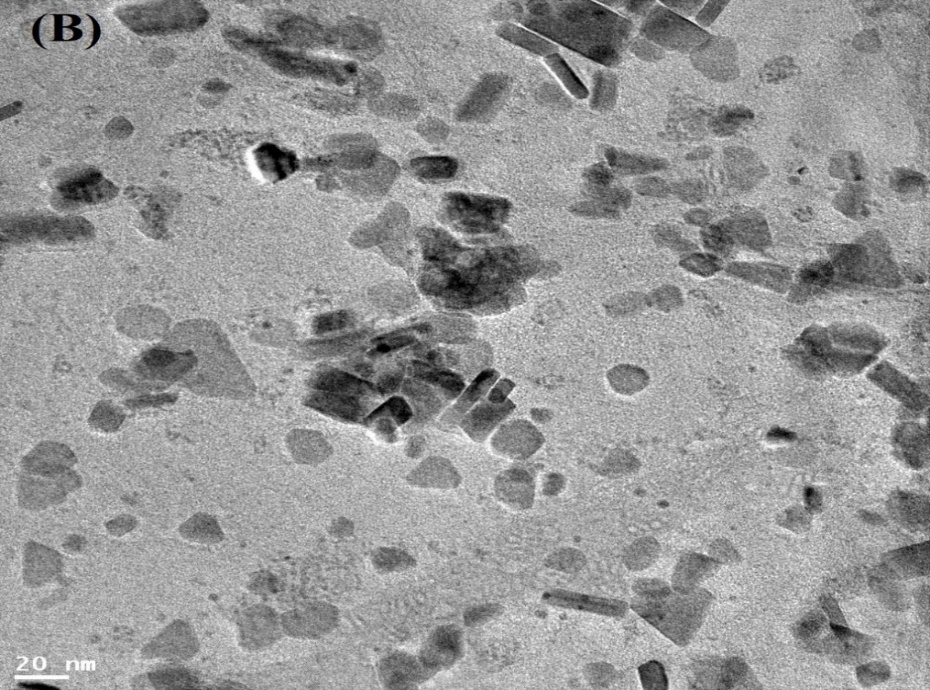 |
|  |
| 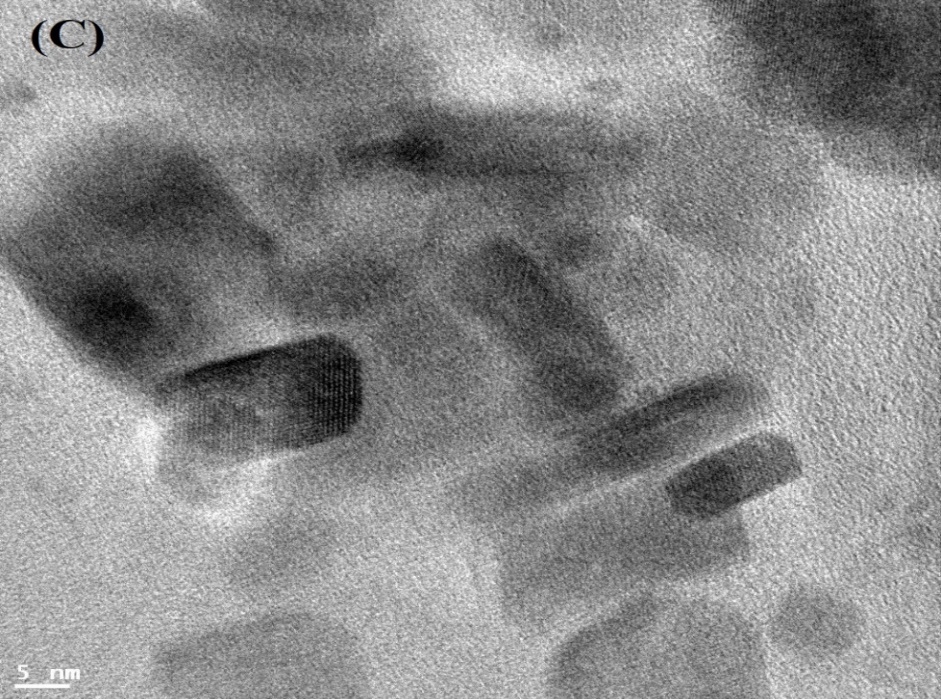 |
|  |
| 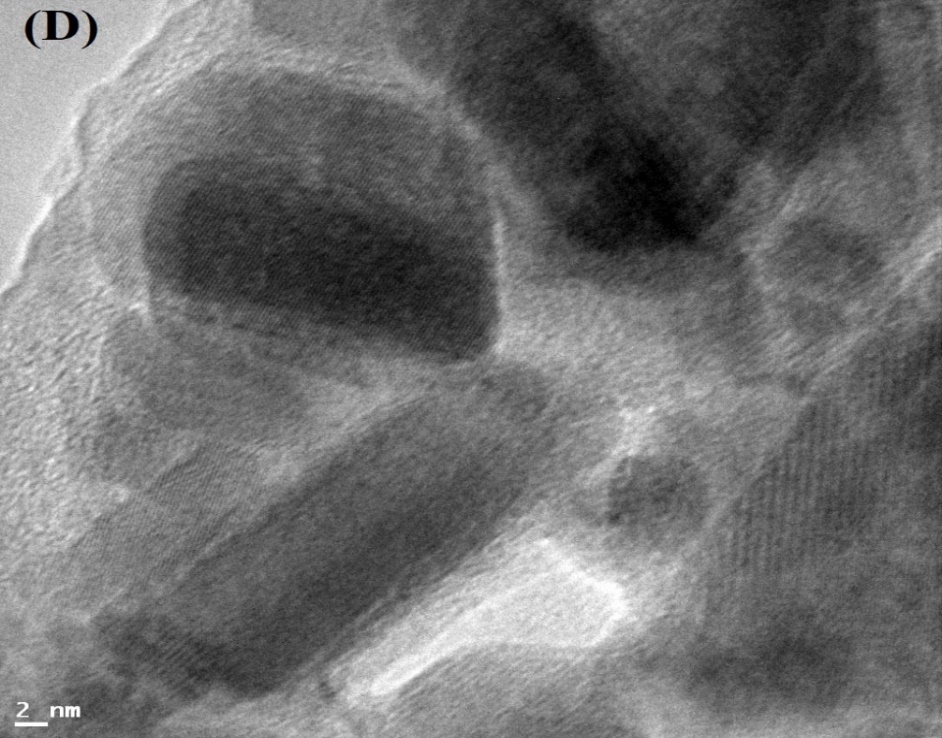 |
|  |

**Fig. S1** TEM images of GO (A) and **SF@GOC** (B, C, D) with different magnification.

***Nitrogen Adsorption-desorption Isotherm Study***

The BET analysis was used to calculate the surface area of the GO and **SF@GOC**. N_2_ adsorption-desorption isotherm type IV was used to characterise the BET analysis for the produced composite (Figure S2). The isotherm showed a significant hysteresis loop at high relative pressure and excellent adsorption with thin slit holes. The distribution of pore size revealed that its range was rather constrained. In comparison to 132.54 m^2^ g^-1^ of GO surface area, the composite **SF@GOC** possessed 188.05 m^2^ g^-1^ of well-developed BET specific surface area, 9.159 cm^3^/g of total pore volume, and 6.7 Å as average pore diameter calculated from the matched nitrogen isotherm for **SF@GOC** compared to GO ie.13.013 cm^3^/g pore volume and 11.0 Å pore diameter. The results demonstrated that the surface area increased as a result of the altered **SF@GOC** structural properties (Table 1). This outcome could be explained by the creation of additional active sites, which resulted in increased specific surface area and improved adsorption performance (Baskan and Hadimlioglu, 2021).

|  |
| --- |
|  |

**Figure S2.** N_2_ adsorption/desorption isotherms of GO and **SF@GOC**

**Table S1.** Surface and pore characteristics of **GO** and **SF@GOC**

| Compound | BET surface area (m^2^/g) | Total pore volume (cm^3^/g) | BJH pore diameter (Å) | | |
| --- | --- | --- | --- | --- | --- |
| GO | 132.54 | 13.103 | | 11.0 |  |
| SF-GOC | 188.05 | 9.159 | | 6.7 |  |

**Figure S3:** Mechanistic pathway of degradation of Eosin Y

|  |
| --- |
|  |
|  |
|  |

**Figure S4:** Degradation pathway of Eosin Y

**Figure S5:** Degradation pathway of Orange (II)


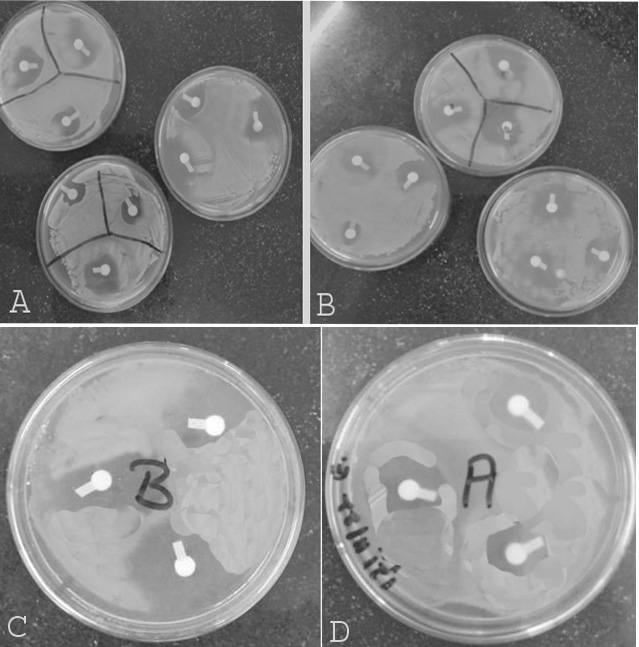


**Figure S6.** The A and B zone of inhibition against 2 mg/ml concentration the Pseudomonas aeruginosa; C and D zone of inhibition against the E. Coli and Staphylococcus strain 10 mg/ml concentration

(a)


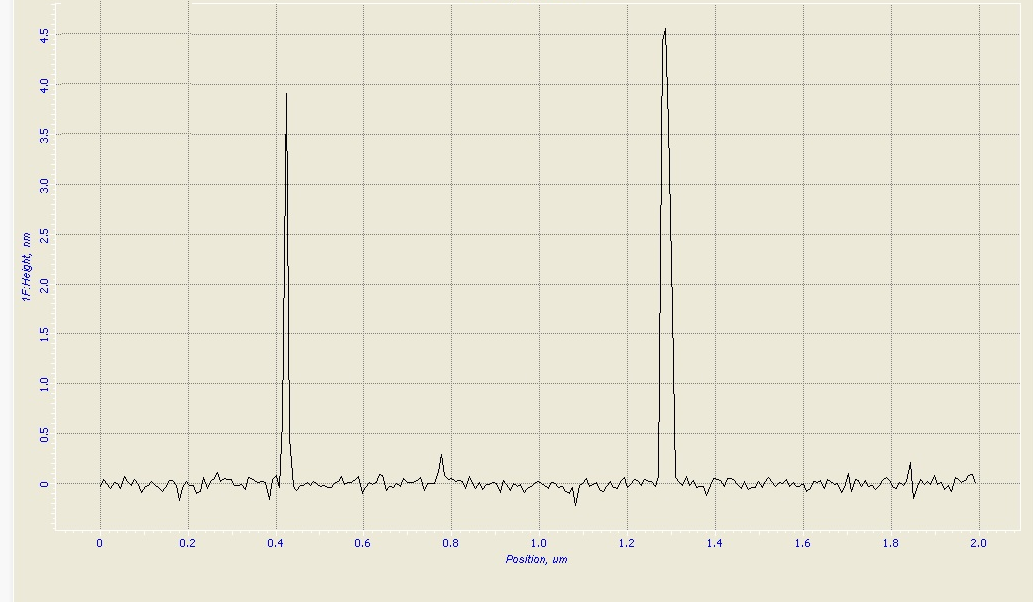


(b)


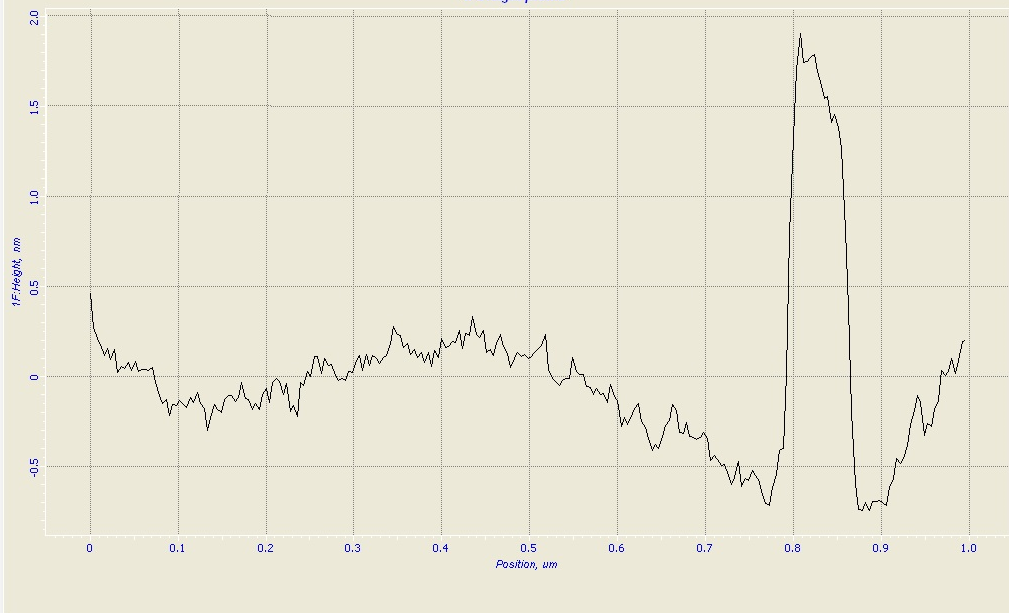


**Figure S7:** Roughness histogram of GO (a) and **SF@GOC** (b) from AFM.

**References**

| Baskan MB and Hadimlioglu S (2021) Removal of arsenate using graphene oxide iron modified clinoptilolite-based composites: adsorption kinetic and column study. J Anal Sc Technol 12:22 |
| --- |
| Kuang L, Liu Y, Fu D, Zhao Y (2016) FeOOH-Graphene Oxide nanocomposites for fluoride removal from water: acetate mediated nano FeOOH growth and adsorption mechanism. J Colloid Interface Sc. http://dx.doi.org/10.1016/j.jcis.2016.11.071 |
